# Navigating the Diagnostic Dilemma: Cecal Duplication Cyst and Intussusception in Pediatric Patients

**DOI:** 10.7759/cureus.105928

**Published:** 2026-03-26

**Authors:** Dimitra Boviatsi, Rafaella Marangou, Thodoris Dadiotis, Nefeli Chaniotaki, Zafeiria G Papathanassiou

**Affiliations:** 1 Department of Radiology, General University Hospital of Patras, Patras, GRC

**Keywords:** cecal duplication cyst, congenital abnormality, diagnosis, intussusception, pediatric patients

## Abstract

Intestinal duplication cyst is a rare congenital anomaly of the digestive tract, which usually affects infants and toddlers. Symptoms are often subtle and nonspecific; thus, a high level of suspicion is critical to a prompt diagnosis. We present a case of cecal duplication cyst in a 17-month-old toddler, who was initially referred to our department with colicky abdominal pain and vomiting. Transabdominal ultrasound indicated intussusception, necessitating barium enema reduction under fluoroscopic guidance. Nevertheless, the fluoroscopic reduction failed, and urgent laparotomy was performed. This report aims to highlight the diagnostic approach and surgical management of this anatomical malformation in pediatric patients.

## Introduction

Intestinal duplication is a sporadic developmental anomaly of the digestive system [1]. According to the current literature, the prevalence is estimated at one in 4,500 live births, with nearly 80% of cases diagnosed before the age of 2 [1,2]. Several scenarios have been proposed regarding the embryological basis of intestinal duplication [1]. The most popular one suggests the entrapment of an embryonic diverticulum or failure of gut recanalization during the fourth to eighth week of gestation [1].

Intestinal duplications are mostly located in the ileum, followed by the jejunum and duodenum, whereas cecal duplications comprise only 0.4% of all gastrointestinal (GI) tract duplications [1,3,4]. They can be either tubular or cystic, with cystic forms being more common [2,5]. Intestinal duplication cysts comprise specific anatomical and histological characteristics, namely, proximity to the mesenteric wall of the GI tract, an outer layer of smooth muscle, and an inner layer of normal GI epithelium [6]. Additionally, duplication cysts often share a common muscle wall and blood supply with the adjacent part of the intestine, more commonly the ileum [2].

Furthermore, GI duplications present with a variety of atypical clinical manifestations and can be complicated by intestinal obstruction, which is often associated with intussusception [6]. Intussusception is a clinical emergency in infancy and early childhood, in which a part of the GI tract invaginates into the more distal segment of the GI tract. It is commonly idiopathic, whereas it can be secondary due to pathological lead points, such as an intestinal duplication cyst [7]. If left untreated, it can result in bowel ischemia and perforation, carrying a dismal prognosis.

We report a rare case of a toddler with intestinal obstruction caused by a cecal duplication cyst. Informed written consent to publish was obtained from the patient’s parents during postoperative hospitalization.

## Case presentation

**Figure 1:**
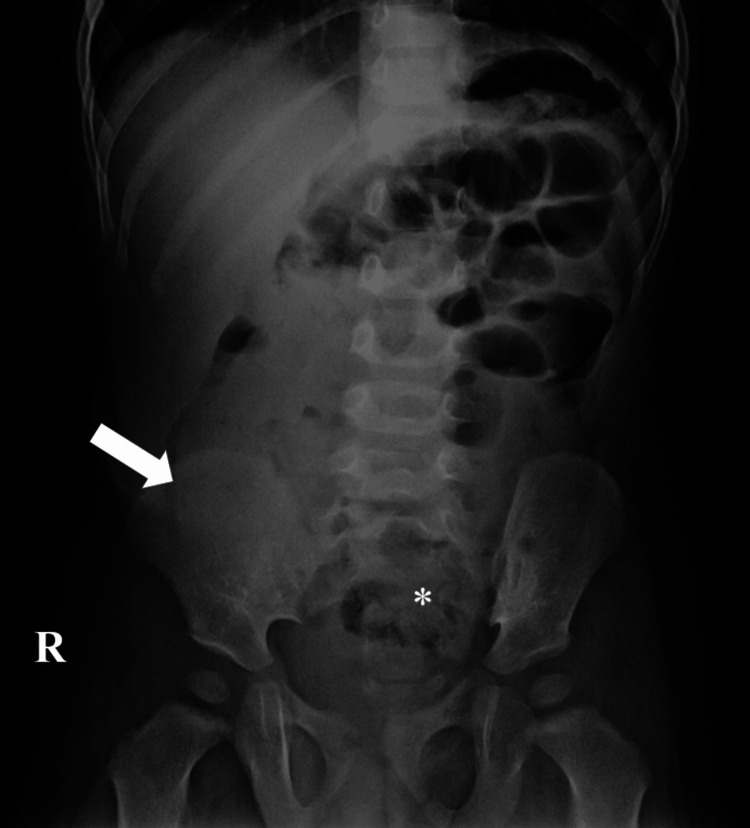
Plain X-ray of the abdomen revealing soft-tissue mass in the RUQ and RLQ Abdominal radiography demonstrates increased radiopacity, especially in the RLQ (arrow), extending toward the hepatic flexure and proximal transverse colon. Distal air in the abdomen is detected (asterisk) R: right; RLQ: right lower quadrant; RUQ: right upper quadrant

A 17-month-old boy was admitted with intermittent acute abdominal pain and four episodes of nonbilious vomiting over the last three days. On thorough clinical examination, mild generalized abdominal tenderness and moderate distension were noted. Plain radiography of the abdomen showed a soft tissue mass in the right upper and lower quadrants, extending toward the hepatic flexure and transverse colon (Figure 1).

**Figure 2:**
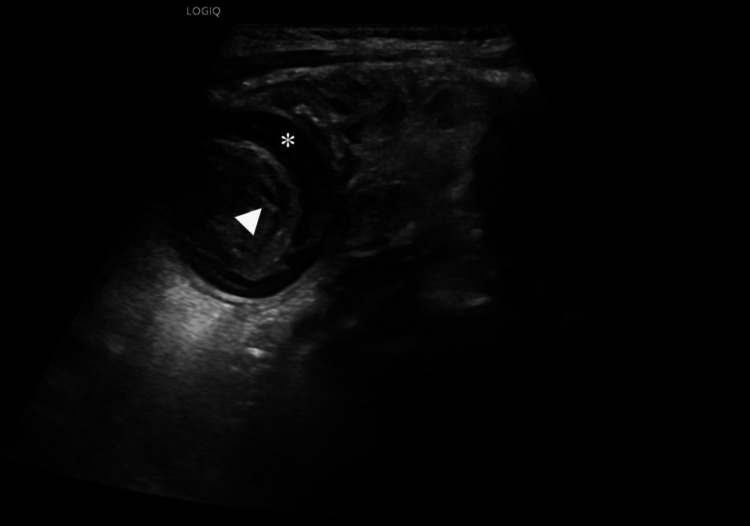
Gray-scale ultrasound image indicating ileocolic intussusception Gray-scale transverse plane reveals a target-like lesion with a hypoechoic rim (asterisk) and hyperechogenic concentric rings (arrowhead), resembling the classic “donut sign”

Subsequent ultrasound assessment revealed a target-like configuration in the right upper abdominal quadrant, raising suspicion of intussusception, while free fluid was detected in the right paracolic gutter (Figure 2).

**Figure 3:**
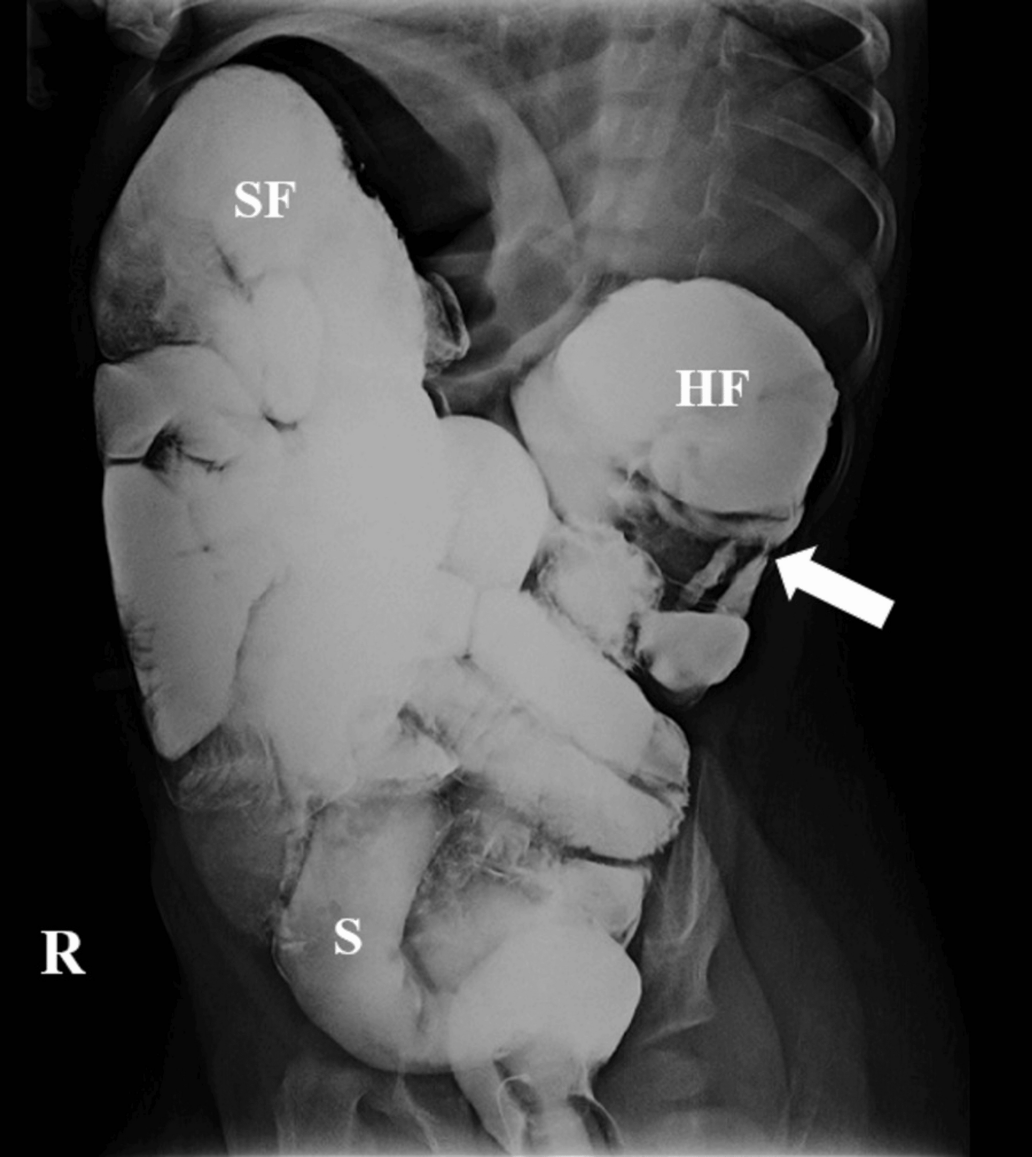
Barium enema study showing partial intestinal obstruction The patient is placed in a right lateral position on the examining table. Contrast passage is identified throughout the rectum, sigmoid, and descending and transverse colon, while minimal contrast is noted proximally to the hepatic flexure (arrow), indicating failed reduction of the intussusception HF: hepatic flexure; R: right; S: sigmoid; SF: splenic flexure

Therefore, a barium enema was administered via a rectal catheter for radiological reduction using fluoroscopy, and minimal contrast passage in the terminal ileum was detected (Figure 3).

**Figure 4:**
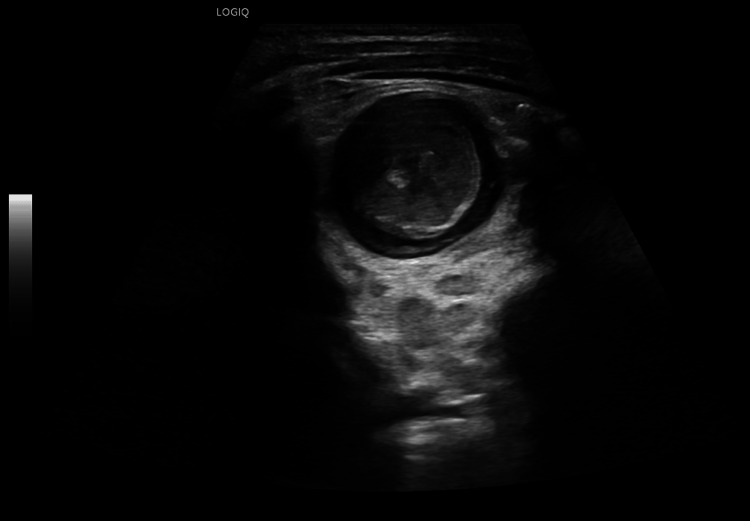
Gray-scale ultrasound plane indicating failure of the enema reduction Follow-up gray-scale transverse image confirms the presence of the target-like configuration

Nevertheless, a second-look ultrasound still showed the target-like lesion with a hyperechoic center (Figure 4).

**Figure 5:**
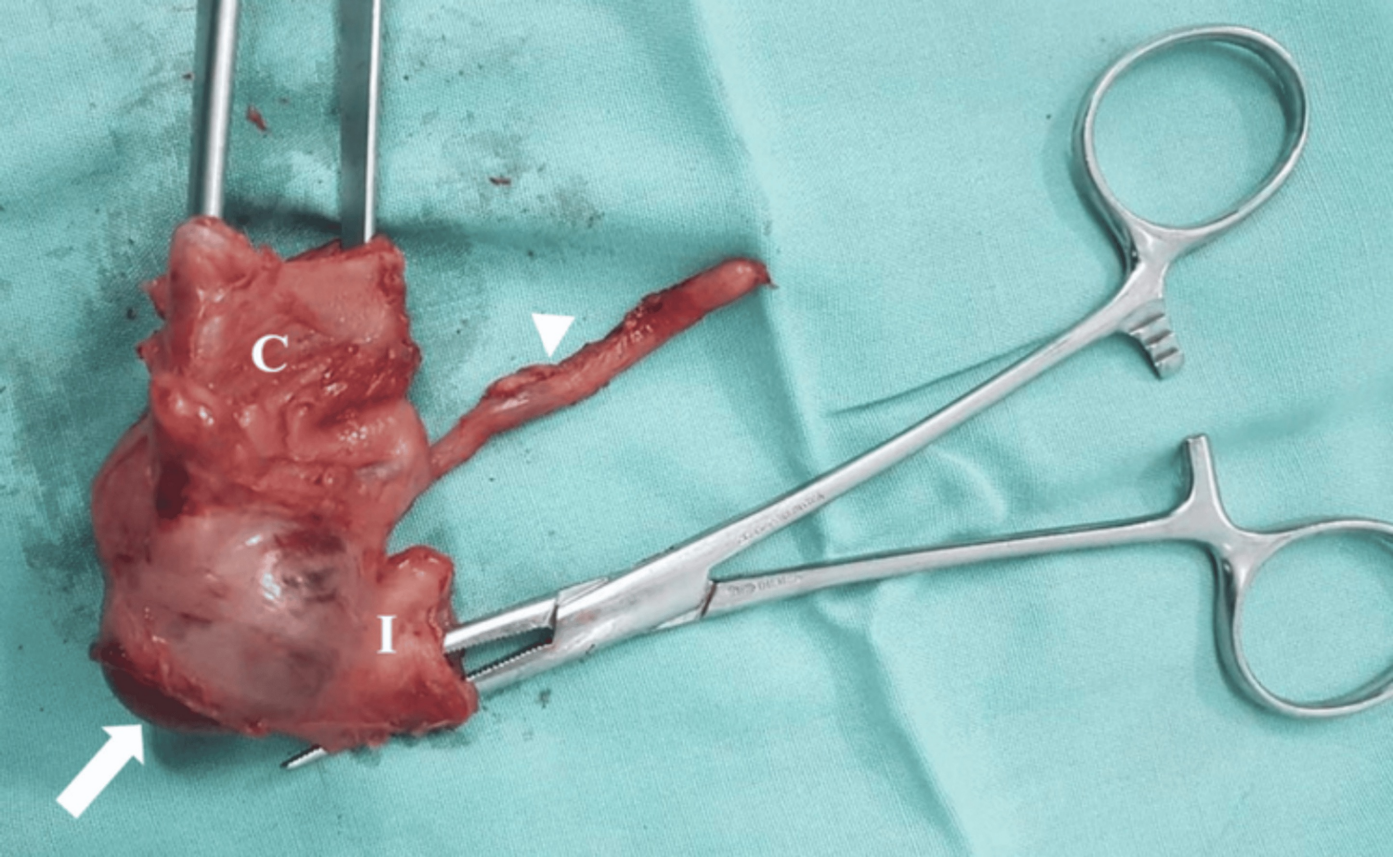
Intraoperative view of the gross specimen No evidence of intussusception was identified, while the appendix appeared normal (arrowhead). An intramural cyst was noted laterally to the ileocecal valve (arrow) C: cecum; I: ileum

The patient underwent urgent laparotomy, and an intramural cecal duplication cyst was identified without evident intussusception. As a result, a right hemicolectomy along with segmental resection of the terminal ileum was performed, and end-to-end anastomosis was conducted (Figure 5).

**Figure 6:**
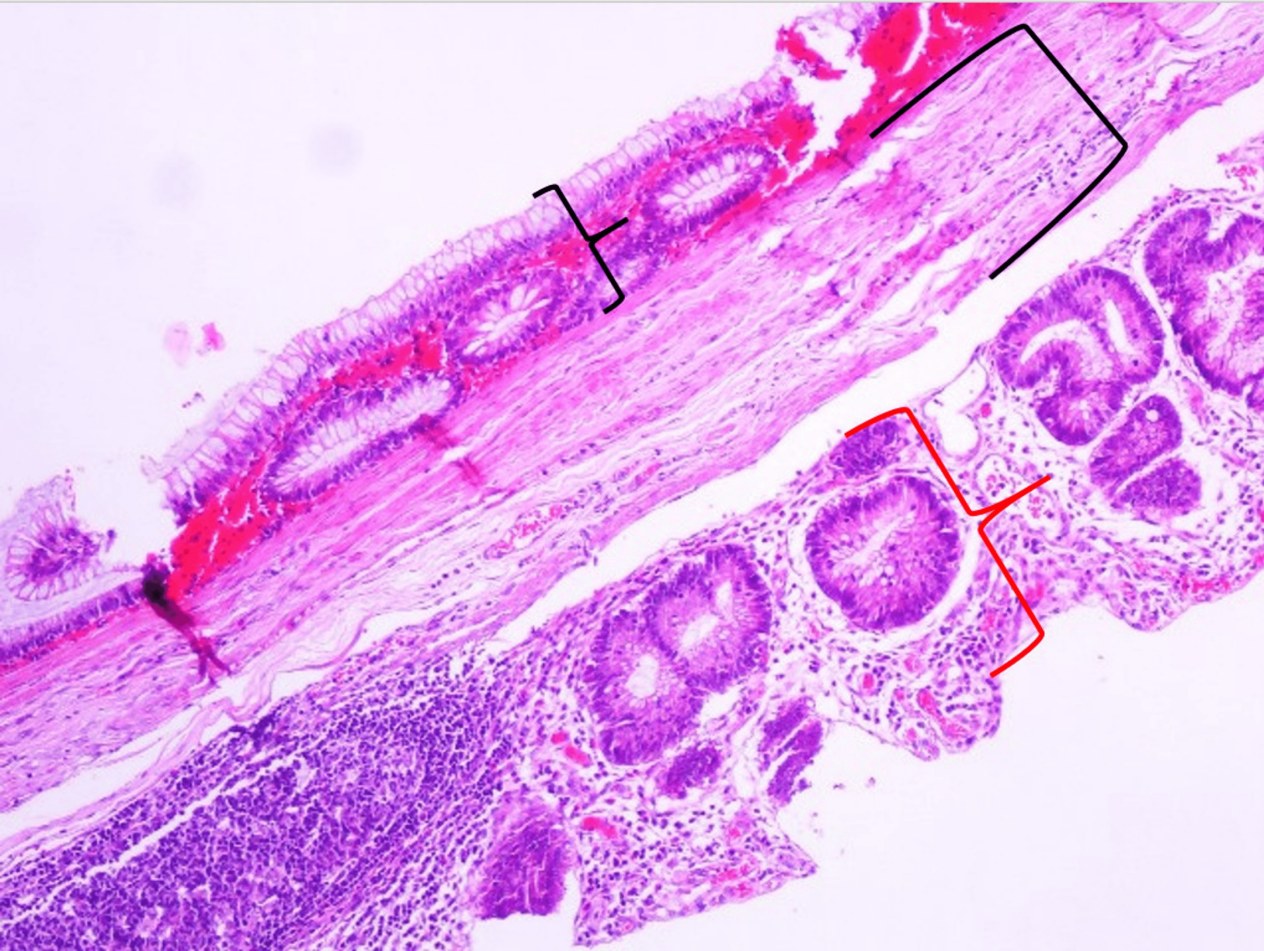
Histopathological image of the dissected cecal cyst and adjacent cecum in 10× microscopic view Photomicrograph (original magnification, 10×; H&E) demonstrates the mucosa of the cecal duplication cyst (black cony bracket), the mucosa of the cecum (red cony bracket), as well as the shared muscular layer in the middle (square bracket) H&E: hematoxylin and eosin

The toddler’s intraoperative and postoperative course was uneventful. Finally, the resected specimen was subjected to morphometric and histopathological examination, revealing a cecal duplication cyst of 3.5 x 3.2 cm, while the microscopic examination after hematoxylin and eosin staining disclosed that the cyst was filled with mucous material (Figure 6).

## Discussion

Duplication cysts can be detected anywhere in the GI tract from the mouth to the anus, with colonic cysts accounting for approximately 13% of cases [2]. According to studies by Schalamon et al. and Radhakrishna et al., intestinal duplications usually affect females, whereas a predominance of male occurrence has been documented [1,6,8,9].

Various theories about the embryological origin of intestinal duplication cysts have been proposed. The main hypothesis postulates that intestinal duplications originate from the entrapment of an embryonic diverticulum or failure of recanalization of the obliterated gut during embryonic development, causing segmental duplication without concurrent congenital abnormalities [9]. On the other hand, the theory by Bentley and Smith [10] holds that intestinal duplications are attributed to abnormal adhesions of the ectoderm and endoderm, followed by herniation of the yolk sac between the anterior and posterior half of the embryonic vertebrae, namely, split notochord syndrome [1]. Additional co-existing malformations, such as spinal deformities, also support this embryological hypothesis [1]. However, no consensus has been achieved so far regarding the exact pathogenesis.

Duplication cysts can be either asymptomatic or cause a variety of symptoms depending greatly on their size and location, including abdominal pain, distension, vomiting, constipation, and diarrhea [1,2]. Abdominal pain can be associated with the accumulation of secretions within the cyst, compressing or obstructing the adjacent bowel segment, and complications such as intussusception [3,4]. Occasionally, duplication cysts can present with GI bleeding, as ectopic gastric mucosa has been detected in one-third of cases, predisposing to ulceration [1,2]. Intestinal obstruction, intussusception, volvulus, infection, perforation, and malignancy are possible complications of a duplication cyst [3]. However, malignant transformation of duplication cysts is extremely rare in children, primarily in colonic duplications [2,3]. Furthermore, duplication cysts are associated with congenital anomalies, affecting mostly vertebrae, such as hemivertebrae, spinal column duplication, and spina bifida, as reported by Schalamon et al. [1]. Urogenital anomalies, such as duplication of the urinary and genital organs and ovarian cysts, have also been described in cases of GI duplication in a previous study by Otter et al. [5]. Additional documented abnormalities include mental retardation, hydrocephalus, meningocele, hernias, bilobar right lung, Meckel's diverticulum, hip dysplasia, and club foot [5]. However, no concurrent congenital abnormalities were observed in our case.

Nevertheless, clinical manifestations of intestinal duplication cysts are atypical, mimicking other intra-abdominal entities such as intussusception, volvulus, appendicitis, mesenteric, and ovarian cysts [11]. Intussusception constitutes an urgent condition, causing intestinal obstruction in infants and children [12]. Although it is usually idiopathic due to lymphoid hyperplasia of the terminal ileum, pathologic lead points should be considered, especially in children over five years old, such as Meckel’s diverticulum, intestinal duplication cyst and polyp, hamartoma, and malignant lymphoma [7,13]. Patients present with typical symptoms, including colicky abdominal pain, vomiting, and red currant stools, in less than 50% of cases [14]. Hence, a prompt diagnosis is established by imaging studies [7].

A multimodal approach to intestinal duplication cysts is crucial, as it can easily be misdiagnosed or even overlooked in patients with subtle symptoms. A plain X-ray of the abdomen is a first-line imaging study, showing a soft tissue mass with or without signs of GI obstruction, depending on the size and location of the lesion [4,8]. Supplementary contrast studies, such as an upper GI series with small bowel follow-through or an enema examination using barium or water-soluble contrast media, can show a submucosal mass extending into the GI tract, providing useful information regarding the level and degree of obstruction [3].

Ultrasonography constitutes a reliable imaging modality for diagnosing an intestinal duplication cyst with 95% specificity and a positive predictive value (PPV) of over 90%, according to a study by Khalid et al. [11]. Furthermore, it is considered the imaging modality of choice regarding the diagnosis of intussusception, with a high specificity and PPV of over 95% and 85%-100%, respectively [15]. In cases of intestinal duplication cysts, ultrasound demonstrates a double-layered wall appearance of the cyst, namely, a hyperechogenic inner mucosal layer and a hypoechoic outer muscular layer [3,6]. This double-layered appearance is a key sonographic finding, presenting in more than half of the cases [3]. However, the layers of the duplication cyst may be nonconcentric, as they are often asymmetrical in thickness [3].

Regarding intussusception, transabdominal ultrasound can reveal the classic “target sign,” which consists of a rim of multiple concentric rings around an echogenic center measuring above 2.5 cm in diameter in a transverse plane [16,17]. A “donut sign” can also be detected in cases of intussusception, representing a thick hypoechoic rim of edematous distal ileum, greater than 10 mm in anteroposterior diameter [16,17]. Additional findings include the presence of trapped intraperitoneal fluid, mesenteric lymph nodes, or fat in the target-like mass [18]. In our case, a hyperechoic central portion of the target-like lesion was noted, resembling intussuscepted mesenteric fat, which proved to be an intramural cyst filled with mucinous content. Although multiple studies have demonstrated a high diagnostic accuracy of ultrasound regarding intussusception, a radiologist should consider an alternative diagnosis if sonographic findings persist after radiological reduction, as documented in previous studies by Radhakrishna et al. and Karakus et al. [9,15,19].

Cross-sectional imaging studies are rarely used in pediatric patients for diagnosing an intestinal duplication cyst, although they can be useful in complicated cases that necessitate multiplanar visualization of the lesion [6]. Nevertheless, advanced imaging studies, including CT and MRI, are not preferred, as contrast medium administration and sedation are required, while exposure to ionizing radiation cannot be avoided in CT imaging.

Surgical resection is the definitive treatment of an intestinal duplication cyst, aiming to avoid further complications, especially in symptomatic patients [4,9]. Surgical treatment is also recommended when an intestinal duplication cyst constitutes an incidental imaging finding [4]. Total excision with resection of the adjacent GI segment and end-to-end anastomosis is the first-line treatment [4,9]. On the other hand, radiological reduction is the preferred treatment in cases of intussusception, given the absence of contraindications, such as perforation or signs of hemodynamic instability [7,13]. This method includes a pneumatic or hydrostatic enema guided by fluoroscopy or ultrasound, which is successful in about 85%-90% of cases [20]. However, intussusception induced by lead points, such as duplication cysts, is correlated with multiple recurrences as well as failure of radiologic reduction [7,13]. If the intussusception cannot be resolved after three attempts, surgical intervention is required [17]. Moreover, an ultrasound examination after each radiologic reduction attempt should be included in the diagnostic algorithm to evaluate for the presence of intussusception and identify the underlying cause. In our case, a target-like lesion was detected on follow-up ultrasound after enema reduction, which was attributed to a cecal duplication cyst intraoperatively.

## Conclusions

Intestinal duplication cysts should be considered in the differential diagnosis of intermittent abdominal pain in infants and children. Ultrasound is a well-tolerated, cost-effective, and accessible imaging method and follow-up approach for pediatric patients with suspected intussusception. However, a radiologist should be mindful of entities resembling the "target sign" of intussusception, such as a cecal duplication cyst, while a second-look ultrasound following radiological reduction should be included in the diagnostic algorithm. Given its high diagnostic value, ultrasound should be the first-line imaging modality, as timely diagnosis of an intestinal duplication cyst is critical to avoid complications and unnecessary interventions, and improve postoperative outcomes. Nevertheless, further studies are warranted to better define diagnostic criteria and optimize surgical management.
